# Susceptibility testing of *Leishmania* spp. against amphotericin B and fluconazole using the Sensititre™ YeastOne™ YO9 platform

**DOI:** 10.1186/s12879-019-4237-3

**Published:** 2019-07-08

**Authors:** Ruwandi Kariyawasam, Priyanka Challa, Rachel Lau, Andrea K. Boggild

**Affiliations:** 1 Institute of Medical Sciences, University of Toronto, Address: 1 King’s College Circle, Toronto, Ontario M5S 1A8 Canada; 2 Faculty of Arts and Science, University of Toronto, Address: 100 St. George Street, Toronto, Ontario M5S 3G3 Canada; 3 Public Health Ontario Laboratory, Address: 661 University Avenue, Toronto, Ontario M5G 1M1 Canada; 4 Department of Medicine, University of Toronto, Toronto, Ontario Canada; 5 Tropical Disease Unit, Toronto General Hospital, Toronto, Address: 200 Elizabeth Street, 13EN-218, Toronto, ON M5G 2C4 Canada

**Keywords:** *Leishmania*, Drug susceptibility, Sensititre™ YeastOne™ YO9, Amphotericin B, Fluconazole, Azole antifungals

## Abstract

**Background:**

Current drug regimens for cutaneous leishmaniasis (CL) include toxic systemic therapies such as amphotericin B (AB) and pentavalent antimonials. Fluconazole (FZ) is a well-tolerated potential oral alternative for the management CL. To date, few objective data exist to guide clinical decision-making when selecting a therapeutic agent a priori, and standardized, clinically-approved drug susceptibility testing platforms for *Leishmania* spp. have yet to be established. The Sensititre™ YeastOne™ YO9 plate is a commercialized drug susceptibility plate including AB and FZ used for routine testing of non-fastidious yeast. Our objective was to adapt the readily available Sensititre™ YeastOne™ YO9 plate, to determine drug susceptibility profiles of AB and FZ in cultured isolates of Old World and New World *Leishmania* spp. for the treatment of CL.

**Methods:**

Promastigotes were cultured in Tobie’s medium with Locke’s overlay until log phase growth was achieved, inoculated into the Sensititre™ system, and incubated over 96 H*. minimum* inhibitory concentrations (MICs) were determined colorimetrically, and promastigote death was assessed by conventional microscopy out to 96- h. Colour change correlated to MIC values.

**Results:**

All strains tested exhibited MIC values for FZ that were ≥ 256 μg/mL. New World strains demonstrated reduced susceptibility to AB (0.25 μg/mL – 0.50 μg/mL AB) compared to Old World strains at 0.12 μg/mL AB (*p* = 0.02). Seventeen (61%) of 28 *Viannia* isolates versus 82% (27/33) of non-*Viannia* isolates were resistant at 0.12 μg/mL AB (*p* = 0.09). For *L. V. braziliensis* isolates, mean MIC for AB was 0.375 ± 0.14 μg/mL (range 0.25–0.50 μg/mL), while for isolates of *L. V. panamensis* it was 0.314 ± 0.26 μg/mL (range 0.12–1.0 μg/mL).

**Conclusions:**

We adapted the Sensititre™ YeastOne™ YO9 plate for testing of *Leishmania* spp*.* susceptibility profiles for commonly used antifungals in the treatment of CL, including AB and FZ. Given its current utility in mycology, optimization of the system for potential clinical implementation in parasitology should be pursued. However evaluation of clinically relevant amastigote-stage stages, and higher concentrations of FZ beyond the upper limit concentration of the Sensititre™ YeastOne™ Y09 plate would be required.

## Background

Leishmaniasis, a parasitic disease caused by the protozoan *Leishmania*, affects millions of the world’s poorest, and is ranked among the top three most common travel acquired dermatoses [1, 2]. Parenteral drugs available in North America include formulations of Amphotericin B (AB), pentavalent antimonials and pentamidine, while oral drugs, including miltefosine and azole antifungal compounds, are options for oral treatment of CL [3]. Pentavalent antimonials, including sodium stibogluconate and meglumine antimoniate, are regarded as first-line treatment against New World CL [2–4]. This class of drugs is highly toxic, often difficult to access, and requires enhanced clinical monitoring or hospitalization to prevent irreversible toxicities to the heart, liver, kidney, and pancreas [2–4]. Clinically significant adverse events, including but not limited to, severe thrombocytopenia and pancreatitis, are common reasons for treatment interruption with antimonials. Miltefosine is a highly effective oral alternative to antimonials for the treatment of CL [3], though its use is limited by prohibitively high cost. Amphotericin B (AB) is a polyene antifungal that targets the sterol rich membranes of *Leishmania* spp. by producing ion-channel pores spanning the lipid bilayer, and increasing cell membrane permeability to small ions and solute molecules, resulting in cell death [5, 6]. Four formulations of AB, including amphotericin B deoxycholate, liposomal amphotericin, cholesterol dispersion amphotericin, and lipid complex amphotericin are used as second-line treatment of CL, and vary in treatment efficacy [3, 5–8]. Severe side effects of AB, especially renal toxicity are mostly associated with amphotericin B deoxycolate compared to other amphotericin formulations [3, 5–8].

Antifungal azoles such as itraconozole, ketonazole, and fluconazole (FZ), have been evaluated in clinical trials of CL treatment, and species-specific results suggest that these comparatively well-tolerated oral regimens also demonstrate efficacy, particularly for Old World CL [3, 4, 9–11]. FZ inhibits C14α-demethylase of the ergosterol biosynthetic pathway [7]. The oral formulation, long half-life, and high skin-to-plasma concentrations make it a popular alternative in the treatment of CL [9]. Widespread use of FZ for CL treatment is limited by a lack of large randomized controlled trials demonstrating efficacy, and the need for high-dose administration in order to achieve cure [3, 9–11]. Current clinical management guidelines indicate that “no ideal or universally applicable therapy for CL has been identified”, and that selection of therapy should be individualized [3]. Given the absence of a first-line agent and considering the many factors such as patient preference, lesion localization, cost, ease of administration, probable efficacy, likelihood of subsequent mucosal disease, age, existing co-morbidities, and drug accessibility, treatment should be individualized, and this process would be enhanced by drug susceptibility platforms to inform clinical decision-making [3]. Geographic and anticipated species-specific response to therapy should also be considered when selecting a therapeutic agent, particularly given high rates of failure of antimonials in pockets of endemicity [3, 12, 13]. However, few objective parasitologic data exist to guide the clinical decision-making process when selecting a therapeutic agent a priori. Estimating the likelihood of drug failure is largely informed by physician experience and clinical and epidemiological data, rather than objective parasitologic metrics, as one would achieve through standardized in-vitro drug susceptibility testing (as is done for countless other microbial infections).

At present, in-vitro systems for assessing predominantly Old World strains of *Leishmania* spp*.* susceptibility include: agar dilution, broth microdilution, flow cytometry, reporter gene assays, enzymatic determination, H^3^-thymidine incorporation, and colorimetric assays including the use of resazurin based Alamar Blue [14–18]. These techniques are primarily used in research laboratories, and have yet to be validated for routine clinical use due to their time-consuming nature, and requirement of substantial technical expertise and laboratory infrastructure. Another challenge to routine drug susceptibility testing is the biphasic life cycle of *Leishmania*, where the larger, motile (and therefore more visible by light microscopy) promastigote stage inhabits the sandfly midgut, while the 2-μm amotile amastigote resides inside the mammalian host macrophages, where it evades immune detection. Intracellular amastigotes harvested from macrophages remain the gold standard for testing drug susceptibility in-vitro [14]. However, given that log-phase promastigotes are generally more resistant to anti-*Leishmania* drugs than amastigotes [14–17], and detectable in a cell-free culture system incubated at room temperature, promastigotes are surrogates of an isolate’s susceptibility pattern, independent of cell-mediated parasiticidal mechanisms [14].

The Sensititre™ YeastONE™ YO9 Susceptibility Plate (Thermo Scientific), used for routine quantitative antifungal susceptibilities (MIC) in non-fastidious yeast, such *Candida* spp. and *Cryptococcus* spp., contains the following antifungals: anidulafungin, amphotericin B, micafungin, caspofungin, 5-flucytosine, posaconazole, voriconazole, itraconazole and fluconazole [19]. The alamarBlue® technology is a colorimetric growth indicator based on detectable metabolic activity, and remains constant with extended incubation times (as are required for culture of *Leishmania*), and across inoculation media [19, 20]. Thus, it could be adapted for use in *Leishmania* susceptibility testing within an antifungal-based panel, such as the Sensititre™ YeastOne™ YO9 Susceptibility Plate.

Given the scarcity of well tolerated, easily accessible, and inexpensive therapies coupled with the necessity to treat active lesions of Latin American *Viannia* strains to potentially minimize the risk of downstream mucosal disease, as well as the propensity of strains to fail all available therapies with sufficient frequency, a user-friendly drug susceptibility testing platform with potential for clinical implementation should be developed. In order to address several aspects of this existing knowledge and care gap, we adapted the Sensititre™ YeastONE™ YO9 Susceptibility Plate to examine AB and FZ susceptibility profiles in log-phase *Leishmania* spp. promastigotes. We herein report the use of this commercialized antifungal drug susceptibility platform as proof-of-concept to assess drug susceptibility profiles of *Leishmania* strains imported to Canada and available via the American Type Culture Collection (ATCC®).

## Materials and methods

### Parasite cultures

ATCC® and clinical cultures submitted to the Public Health Ontario Laboratory for *Leishmania* spp*.* diagnostic testing were routinely sub-cultured in Tobie’s medium with Locke’s overlay (in-house) at ambient temperature every week. The following ATCC© strains were used: *Leishmania Viannia braziliensis* ATCC® 50135™ (MHOM/BR/75/M2903), *L. V. guyanensis* ATCC®50126™ (MHOM/BR/75/M4147)*, L. V. panamensis* ATCC®50158™ (MHOM/PA/71/LS94), *L. amazonensis* ATCC®50159™ (IFLA/BR/67/PH8)*, L. chagasi* Cunha and Chagas ATCC®50133™ (MHOM/BR/74/PP75)*, L. donovani* (Laveran and Mesnil) Ross ATCC®50212™ (MHOM/IN/80/DD8)*, L. infantum* Nicolle ATCC®50134™ (MHOM/TN/80/IPT-1)*, L. major* ATCC®50122™ (MHOM/IL/67/JERICHO II)*, L. mexicana* (Biagi) Garnham ATCC®50157™ (MHOM/BZ/82/BEL21), and *L. tropica* (Wright) Luhe ATCC®50129™ (MHOM/SU/74/K27) (Table 1)*.* The following clinical strains were tested: *L. V. braziliensis* (*n* = 1), *L. V. panamensis* (*n* = 5), *L. infantum* (*n* = 1), and *L. tropica* (*n* = 2) (Table 1).

**Table 1:** Clinical, demographic, parasitological and drug susceptibility data for all strains

Strain	Amphotericin B (μg/mL)	Fluconazole (μg/mL)	Isolate Origin	New or Old World Origin	Clinical Manifestation
*L. amazonensis* ATCC®50159™	0.58 ± 0.22	≥256	Brazil	New World	CL
*L. chagasi* ATCC®50133™	0.33 ± 0.08	≥256	Brazil	New World	VL
*L. donovani* ATCC®50212™	0.25 ± 0.00	≥256	India	Old World	VL
*L. infantum* ATCC®50134™	0.22 ± 0.07	≥256	Tunisia	Old World	VL
*L. major* ATCC®50122™	0.44 ± 0.06	≥256	Israel	Old World	CL
*L. mexicana* ATCC®50157™	0.33 ± 0.08	≥256	Belize	New World	CL
*L. tropica* ATCC®50129™	0.25 ± 0.00	≥256	Former USSR (Azerbaidjanskaya)	Old World	CL
*L. V. braziliensis* ATCC® 50135™	0.25 ± 0.00	≥256	Brazil	New World	CL or ML
*L. V. guyanensis* ATCC®50126™	0.39 ± 0.09	≥256	Brazil	New World	CL or ML
*L. V. panamensis* ATCC®50158™	0.5 ± 0.00	≥256	Panama	New World	CL or ML
Clinical *L. infantum* 1	0.12 ± 0.00	≥256	Italy	Old World	VL
Clinical *L. tropica* 1	0.25 ± 0.00	≥256	Afghanistan	Old World	CL
Clinical *L. tropica* 2	0.25 ± 0.00	≥256	Afghanistan	Old World	CL
Clinical *L. V. braziliensis* 1	0.5 ± 0.00	≥256	Peru	New World	CL
Clinical *L. V. panamensis* 1	0.12 ± 0.00	≥256	Costa Rica	New World	CL
Clinical *L. V. panamensis* 2	0.16 ± 0.04	≥256	Costa Rica	New World	CL
Clinical *L. V. panamensis* 3	0.12 ± 0.00	≥256	Costa Rica	New World	CL
Clinical *L. V. panamensis* 4	0.67 ± 0.17	≥256	Ecuador	New World	CL
Clinical *L. V. panamensis* 5	0.49 ± 0.08	≥256	Costa Rica	New World	CL

### Species identification

*Leishmania* genus 18S real time polymerase chain reaction (PCR) was performed as previously described [21]. Species identification included analysis of the internal transcribed spacer 1 (ITS1), ITS2, cysteine proteinase B (CPB), heat shock protein 70 (HSP70), and mannose phosphate isomerase (MPI) targets by PCR, restriction fragment length polymorphism (RFLP) analysis, and Sanger sequencing [22, 23]. As PCR-RFLP analysis of the ITS1 region can only differentiate *L. V. braziliensis* from the other species within the *Viannia* subgenus (*L. V. guyanensis, L. V. peruviana, L. V. panamensis, L. V. lainsoni*), both PCR-RFLP and sequencing analysis of the CPB, HSP70, MPI and ITS2 regions were used to differentiate species within the *Leishmania Viannia* sub-genus. Purified PCR product was used for Sanger sequencing as per Big Dye protocol (Life Technologies). Sequence products were purified and analyzed using the Applied Biosystems 3130xl Genetic Analyzer. Data were standardized using the Sequencing Analyzer program and the Basic Local Alignment Search Tool (BLAST) engine was used to analyze sequences.

### Drug susceptibility plate

Sensititre™ YeastOne™YO9 susceptibility plates (TREK Diagnostics Systems, West Sussex, UK) were stored at room temperature away from sunlight [19]. Concentrations of impregnated AB ranged from 0.12 μg/mL to 8 μg/mL, whereas FZ ranged from 0.12 μg/mL to 256 μg/mL, as per manufacturer’s documentation (TREK Diagnostics Systems, West Sussex, UK) [19].

### Promastigote assay

Clinical and ATCC® cultures of *Leishmania* spp. promastigotes were maintained in Tobie’s medium with Locke’s overlay at room temperature until log phase growth was achieved. Given that the Sensititre™ YeastOne™ YO9 susceptibility plates are validated for non-fastidious yeasts, *Leishmania* promastigote-specific alterations were made to the inoculum broth, inoculation procedure, incubation, and test reading procedures in the TREK Diagnostic Systems guidelines [19], to ensure successful growth and interpretation of *Leishmania* spp. promastigotes. A solution containing 100 μL of 2.5 × 10^5^ promastigotes/mL in our in-house promastigote broth including Roswell Park Memorial Institute (RPMI) Medium 1640 (1X) (Gibco) with 10% heat-inactivated fetal bovine serum (HI-FBS) (Gibco) and 20 mM HEPES (Fisher Scientific) (10% RPMI) was inoculated into each well, sealed with transparent adhesive film, and placed into an incubator at 27 °C (rather than yeast incubation of 37 °C) in 5% CO_2_. The plates were incubated for up to 96 h. Colour change was assessed by visual acuity (as described in the TREK Diagnostic Systems guidelines [19]) and metrics of promastigote viability were assessed using conventional inverted light microscopy every 24-h to determine an appropriate MIC up until 96 h. Viability metrics included frank motility with propulsion across the microscopic field, as well as stationary flagellar movement and retention of the structural shape of the promastigote. Resistance at a given drug concentration was defined as > 50% promastigote growth, which was determined by individual or group promastigote motility, as well as maintenance of the structural shape in > 50% of promastigotes in each low power field examined.

### Replicates

Each strain was run in biological triplicates, except in the case of *L. major* ATCC®50122™ (MHOM/IL/67/JERICHO II) and *L. V. guyanensis* ATCC®50126™ (MHOM/BR/75/M4147), which included 4 biological replicates, while *L. infantum* Nicolle ATCC®50134™ (MHOM/TN/80/IPT-1) was performed in 5 biological replicates. Two different clinical strains of *L. tropica* were performed in triplicate, yielding 6 data points, whereas 5 different clinical strains of *L. V. panamensis* were performed in triplicate, resulting in 15 data points.

### Controls

*Candida parapsilosis* (ATCC®22019™) with known antimicrobial susceptibility testing (AST) profiles was used as a positive control, and triplicate incubation was performed using the Sensititre™ YeastOne™ YO9 inoculation broth according to TREK Diagnostic Systems guidelines in order to ensure quality of plates [19]. Similarly, ATCC®22019™ was incubated on the Sensititre™ YeastOne™ YO9 platform using the *Leishmania*-adapted 10% RPMI promastigote inoculation broth, as previously mentioned, to assess and control for any MIC changes that may have arisen from a change in inoculation broth. Negative control plates were incubated in the same conditions described above without culture inoculation.

### Statistical analysis

Statistical analyses were conducted using GraphPad Prism 6 version 6.07 software (GraphPad Software Inc). Mean MICs at 96 h were calculated for each strain. Strains were subsequently grouped according to species, probable phenotype, and isolate source and compared using Fisher’s exact test with an alpha or 0.05 and power of 80. Species originating from Latin America were categorized as “New World”, while those originating from Africa, Asia, or the Middle East were categorized as “Old World”. Species known to localize to viscera were classified as “visceralizing” (e.g., *L. donovani, L. infantum, L. chagasi*), while strains causing predominantly tegumentary disease were classified as “non-visceralizing” (*Viannia* strains, *L. major, L. tropica, L. amazonensis, L. mexicana*). MICs of *Viannia* strains, which are known to cause disfiguring mucosal disease, were compared to both non-*Viannia* Latin American strains (e.g., *L. mexicana, L. amazonensis*), and to all non-*Viannia* strains including those originating from the Old World.

## Results

### Promastigote assay

We evaluated AB and FZ susceptibility in 19 strains of clinical and ATCC® isolates from 10 different species of *Leishmania* using the Sensititre™ YeastOne™ YO9 plate. The lowest concentration showing inhibition of growth (i.e., the absence of colorimetric change), correlated to promastigote death, which was assessed by inverted light microscopy at 96 h.

### ATCC®22019™ *C. parapsilosis*

MIC interpretative criteria for *Candida* spp., as per Clinical & Laboratory Standards Institute (CLSI) M27 guidelines, revealed within range susceptibilities for AB (0.83 μg/mL, *n* = 3) and FZ (1 μg/mL, *n* = 3) for ATCC®22019 *C. parapsilosis* following the TREK Diagnostic Systems protocol as per manufacturer’s guidelines [19]. The average MIC value observed for AB and FZ was 1 μg/mL (*n* = 3) after inoculation with the *Leishmania*-adapted 10% RPMI promastigote inoculation broth, falling within range of expected susceptibilities according to the CLSI M27 guidelines, and not differing from the MICs obtained using the standard validated inoculation broth designed for non-fastidious yeasts.

### Fluconazole

MIC values of FZ for all strains of *Leishmania* tested were determined to be ≥256 μg/mL (Table 1). Visible promastigote motility was evident in all FZ-impregnated wells out to 96-h with no colorimetric change in the incubation broth.

### Amphotericin B

Average AB MIC values were observed for the following ATCC® strains: *L. amazonensis* (0.58 μg/mL, *n* = 3), *L. chagasi* (0.33 μg/mL, *n* = 3), *L. donovani* (0.25 μg/mL, *n* = 3), *L. infantum* (0.22 μg/mL, *n* = 5), *L. major* (0.43 μg/mL, *n* = 4*), L. mexicana* (0.33 μg/mL*, n* = 3), *L. tropica* (0.25 μg/mL, *n* = 3), *L. V. braziliensis* (0.25 μg/mL, *n* = 4), *L. V. guyanensis* (0.25 μg/mL, *n* = 4), and *L. V. panamensis* (0.50 μg/mL, *n* = 3) (Table 1). Average AB MIC values were observed for the following clinical strains*: L. infantum* (0.12 μg/mL, *n* = 3*), L. tropica* (0.25 μg/mL, [2 strains, *n* = 3 replicates each for a total of 6 data points), *L. V. braziliensis* (0.5 μg/mL, *n* = 3*), L. V. panamensis* (0.24 μg/mL, [5 strains, *n* = 3 replicates each for a total of 15 data points]) (Table 1).

The following Old World strains were resistant, as defined by > 50% promastigote growth, to AB at 0.12 μg/mL: ATCC® *L. donovani* (*n* = 3, 100%), ATCC® *L. infantum* (*n* = 2, 40%), ATCC® *L. tropica* (*n* = 3, 100%), clinical *L. tropica* (*n* = 6, 100%), and ATCC® *L. major* (*n* = 4, 100%) (Table 2). The following New World strains were resistant to AB at 0.12 μg/mL: ATCC® *L. amazonensis* (*n* = 3, 100%), ATCC® *L. chagasi* (*n* = 3, 100%), ATCC® *L. mexicana* (*n* = 3, 100%), ATCC® *L. V. braziliensis* (*n* = 3, 100%), clinical *L. V. braziliensis* (*n* = 3, 100%), ATCC® *L. V. guyanensis* (*n* = 2, 50%), ATCC® *L. V. panamensis* (*n* = 3, 100%), and clinical *L. V. panamensis* (*n* = 4, 27%) (Table 2). The following strains were resistant to AB at 0.25 μg/mL: ATCC® *L. V. amazonensis* (*n* = 2, 67%), ATCC® *L. chagasi* (*n* = 1, 33%), ATCC® *L. infantum* (*n* = 1, 20%), ATCC® *L. major* (*n* = 3, 75%), ATCC® *L. mexicana* (*n* = 1, 33%), ATCC® *L. V. guyanensis* (*n* = 1, 25%), ATCC® *L. V. panamensis* (*n* = 3, 100%), clinical *L. V. braziliensis* (*n* = 3, 100%), and clinical *L. V. panamensis* (*n* = 3, 25%) (Table 2). The remaining strains were susceptible to AB at 0.50 μg/mL except 1 replicate of ATCC® *L. amazonensis* and clinical *L. V. panamensis*, respectively (Table 2).

**Table 2:** *Leishmania* spp. Antibiogram of ATCC© and Clinical Isolates Resistant at Varying Concentrations of Amphotericin B and Fluconazole

Species	Total Number of Isolates	Amphotericin B (μg/mL)				Fluconazole (μg/mL)
		0.12	0.25	0.5	1	≥256
Old World						
*L. donovani* ATCC®50212™	3	3 (100%)	0 (0%)	0 (0%)	0 (0%)	3 (100%)
*L. infantum* ATCC®50134™	5	2 (40%)	1 (20%)	0 (0%)	0 (0%)	5 (100%)
Clinical *L. infantum*	3	0 (0%)	0 (0%)	0 (0%)	0 (0%)	3 (100%)
*L. tropica* ATCC®50129™	3	3 (100%)	0 (0%)	0 (0%)	0 (0%)	3 (100%)
Clinical *L. tropica*	6	6 (100%)	0 (0%)	0 (0%)	0 (0%)	6 (100%)
*L. major* ATCC®50122™	4	4 (100%)	3 (75%)	0 (0%)	0 (0%)	4 (100%)
New World						
*L. amazonensis* ATCC®50159™	3	3 (100%)	2 (66.6%)	1 (33.3%)	0 (0%)	3 (100%)
*L. chagasi* ATCC®50133™	3	3 (100%)	1 (33.3%)	0 (0%)	0 (0%)	3 (100%)
*L. mexicana* ATCC®50157™	3	3 (100%)	1 (33.3%)	0 (0%)	0 (0%)	3 (100%)
*L. V. braziliensis* ATCC® 50135™	3	3 (100%)	0 (0%)	0 (0%)	0 (0%)	3 (100%)
Clinical *L. V. braziliensis*	3	3 (100%)	3 (100%)	0 (0%)	0 (0%)	3 (100%)
*L. V. guyanensis* ATCC®50126™	4	2 (50%)	1 (25%)	0 (0%)	0 (0%)	4 (100%)
*L. V. panamensis* ATCC®50158™	3	3 (100%)	3 (100%)	0 (0%)	0 (0%)	3 (100%)
Clinical *L. V. panamensis*	15	4 (27%)	3 (25%)	1 (8.3%)	0 (0%)	15 (100%)

**Fig. 1:**
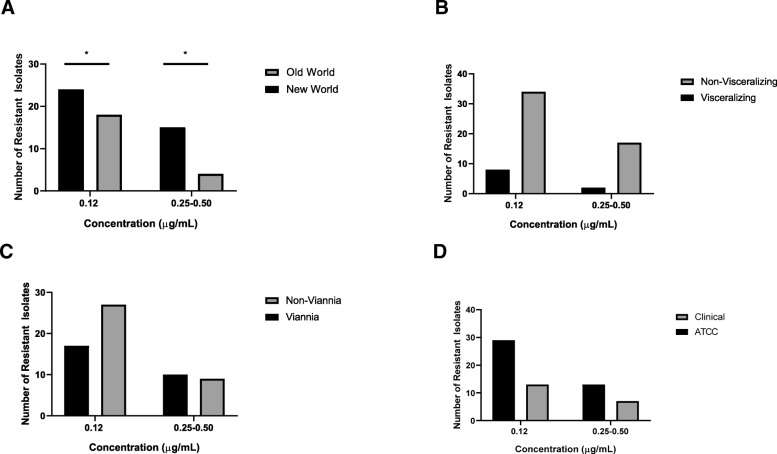
Comparison of the following groups of resistant isolates at 0.12 μg/mL and 0.25 μ/mL-0.50 μg/mL AB by Fisher’s exact test: New World vs. Old World (* *p* < 0.05) (**a**); Visceralizing vs. Non-Visceralizing, (**b**); *Viannia* vs. Non-*Viannia,* (**c**); and, ATCC® vs. Clinical (**d**)

The following isolates demonstrated resistance at 0.12 μg/mL AB: 57% (21/37) of New World isolates versus 88% (21/24) of Old World isolates (*p* = 0.02) (Table 3, Fig. 1a). Eight (57%) of 14 visceralizing isolates were resistant at 0.12 μg/mL AB compared to 72% (34/47) of non-visceralizing isolates (*p* = 0.33) (Table 3, Fig. 1b). Seventeen (61%) of 28 *Viannia* isolates versus 82% (27/33) of non-*Viannia* isolates (*p* = 0.09) were resistant at 0.12 μg/mL AB (Table 3, Fig. 1c). Lastly, twenty-nine (85%) of 34 ATCC® isolates versus 48% (13/27) of clinical isolates (*p* = 0.78) were resistant at 0.12 μg/mL AB (Table 2, Fig. 1d).

**Table 3:** Antibiogram of *Leishmania* spp. Resistance at Varying Concentrations of Amphotericin B and Fluconazole

Species	Total Number of Isolates	Amphotericin B (μg/mL)				Fluconazole (μg/mL)
		0.12	0.25	0.5	1	≥256
Old World						
*L. donovani*	3	3 (100%)	0 (0%)	0 (0%)	0 (0%)	3 (100%)
*L. infantum*	8	2 (25%)	1 (12.5%)	0 (0%)	0 (0%)	8 (100%)
*L. major*	4	4 (100%)	3 (75%)	0 (0%)	0 (0%)	4 (100%)
*L. tropica*	9	9 (100%)	0 (0%)	0 (0%)	0 (0%)	9 (100%)
New World						
*L. amazonensis*	3	3 (100%)	2 (66.6%)	1 (33.3%)	0 (0%)	3 (100%)
*L. chagasi*	3	3 (100%)	1 (33.3%)	0 (0%)	0 (0%)	3 (100%)
*L. mexicana*	3	3 (100%)	1 (33.35%)	0 (0%)	0 (0%)	3 (100%)
*L. V. braziliensis*	6	6 (100%)	3 (50%)	0 (0%)	0 (0%)	6 (100%)
*L. V. guyanensis*	4	2 (50%)	1 (25%)	0 (0%)	0 (0%)	4 (100%)
*L. V. panamensis*	18	7 (39%)	6 (40%)	0 (0%)	0 (0%)	18 (100%)

The following isolates demonstrated resistance at ≥0.25 μg/mL AB (0.25 μg/mL – 0.50 μg/mL): 62% (21/34) of New World isolates versus 78% (21/27) of Old World isolates (*p* = 0.01) (Table 3, Fig. 1a). Two (25%) of 8 visceralizing isolates versus 50% (17/34) of non-visceralizing isolates (*p* = 0.259) were resistant (Table 3, Fig. 1b). Ten (59%) of 17 *Viannia* isolates versus 33% (9/27) of non-*Viannia* isolates were resistant at ≥0.25 μg/mL AB (*p* = 0.12) (Table 3, Fig. 1c). Lastly, thirteen (45%) of 29 ATCC isolates versus 54% (7/13) of clinical isolates (*p* = 0.74) were resistant at ≥0.25 μg/mL AB (Table 2, Fig. 1d).

## Discussion

Treatment of CL is hindered by many factors including, but not limited to: variability in clinical response to treatment with partial correlation to infecting species and region of acquisition; toxicity, expense, and inaccessibility of therapeutics with little pharmacologic innovation over decades; the absence of large-scale therapeutic clinical trials; and the lack of objective laboratory criteria by which to inform likelihood of clinical response and decision-making at the bedside. Clinicians treating patients with CL are provided with little objective parasitologic data to compel selection of one drug over another, and must present patients with clinical guidelines that incorporate a number of contingencies into the decision-making process. Azole antifungals are easily accessible, inexpensive, well tolerated, and supported by several reported trials of efficacy [3, 9], but clinical response to this class of medication can be highly variable compared to other systemic options such as amphotericin and miltefosine, which are more toxic. Development and validation of an objective drug susceptibility system to approximate probable clinical response to therapy should be encouraged, and we have herein demonstrated, as proof-of-concept, that the Sensititre™ YeastOne™ YO9 system is potentially adaptable for routine clinical laboratory testing of AB susceptibility in clinical strains of *Leishmania*. Adaptation of an existing commercialized system guarantees a standard of quality assurance associated with GCP/GLP manufacturing processes, while reducing errors of reproducibility and accuracy compared to systems developed on a smaller, ad hoc, non-commercial scale, as in many research laboratories [14–19]. As per TREK Diagnostic Systems, the Sensititre™ YeastOne™ YO9 system provides results within 96 h from inoculation to final reading of MICs [19]. The clinical utility of this plate is highlighted by its cost-effectiveness, time efficiency, and low burden of technical expertise required. The cost per plate including 9 dehydrated drugs with varying concentrations and media is $30 USD with a maximum of 2 h of technical support to set-up and read the plate. In comparison, other in-vitro platforms require individual drug procurement, which translates to well over $300 USD excluding reagents [14–19]. Moreover, such ad hoc investigational systems lack a standard of quality assurance inherent to commercialization and licensure, and are subject to cross contamination [14–19]. Additionally, time for technical support including experimental set-up, monitoring and reading of plates exceeds 2 h, and requires additional training for outcomes measured by flow cytometry, fluorescence-activated cell sorting (FACS), electron microscopy, zone of inhibition (ZI) analysis, and motile cell counts for disk diffusion and broth dilution methods, respectively [14–18]. Lastly, final MIC readings in such ad hoc systems often exceed 96 h, and, in some cases, require up to 20 days [14–18]. Overall, the Sensititre™ YeastOne™ YO9 system provides a more efficient and objective measure of analysis, which can be compared between laboratories. Other advantages include the less labor intensive technologies such as the plate-impregnated alamarBlue® technology, which eliminates the need for microscopy.

Clinical case reports and studies have demonstrated the efficacy of high-dose FZ in the treatment of CL in both New and Old World strains of *Leishmania* [3, 9–11]. A study conducted in Saudi Arabia demonstrated a 79% cure rate of CL due to *L. major* at 12-weeks following initiation of 6-weeks of 200-mg daily FZ [10]. In another trial, treatment of localized CL due to *L. major* with FZ at 400 mg/day for 42 days led to an 81% cure rate at 6-weeks [11]. In a study of CL due to *L. V. braziliensis,* clinical cure was observed more rapidly and to a greater extent (mean duration of treatment 4-weeks; 100% cure) when FZ was prescribed at 8-mg/kg per day compared to a lower dose 5-mg/kg/day regimen (mean duration of treatment 7.5-weeks; 75% cure) [9]. Recent data surrounding the treatment of *L. V. braziliensis* and *L. V. guyanensis* from the Brazilian Amazon demonstrate sub-optimal cure rates with FZ 6.5–8 mg/kg/day for 28 days and 450 mg/day for 30 days, respectively [24–26], thus reinforcing the need for a standardized objective marker of expected clinical response, one component of which could be a drug susceptibility testing system that would be functional across species and geographic origins. The isolates tested in our study exhibited FZ MICs ≥256 μg/mL in every case, corroborating the observed clinical requirement of high concentrations of FZ for leishmanicidal effect. Further testing of clinical and ATCC® strains of *Leishmania* against higher concentrations of FZ will be important to determine the adaptability of an in vitro system for FZ susceptibility testing.

Liposomal amphotericin B is a less toxic alternative to antimonial treatment of CL and mucosal leishmaniasis (ML), however, further studies on the optimal dosage are required given species-specific cure rates and variation in reports of total treatment dosages [26–28]. Currently, treatment of CL or ML caused by *L. V. brazilienisis* with AB requires a minimum 3 mg/kg dosage over many days [26, 29, 30]. MIC values obtained in this study support average parasiticidal concentrations of 0.5 μg/mL for AB, which equates to a total dose below the recommended dosing schedule, suggesting that a lower, less toxic dose of AB might be effective clinically. Further testing to determine serum blood concentrations to correlate in-vitro drug susceptibility to clinical dosing of AB in cases of CL is necessary and warranted [26, 30].

AB has been proven effective in low dosages against the promastigote form of the parasite in visceralizing species, such as *L. donovani* [26, 29–31]*,* and this clinical phenomenon is reflected in our data as well, given that a greater proportion of non-visceralizing strains demonstrated growth at higher concentrations of AB compared to visceralizing strains. Although we did not observe any differences in actual MIC values to AB, the proportionate growth of visceralizing compared to non-visceralizing isolates highlights a potential trend that is supported clinically, and will be further validated using amastigotes in the next phase of this work.

## Limitations

We graded promastigote viability subjectively by colorimetry and assessment of motility. However, the potential influence of this subjectivity would be mitigated by the use of an objective tool such as the Sensititre™ Vizion™ Digital MIC Viewing System, which would enable easy detection of color changes. Assessment of parasite viability by motility would not exclude the possibility of immotile, metabolically active parasites that could theoretically break-through or rebound in growth following drug cessation. However, given the absence of any clinically-validated susceptibility system for *Leishmania*, this preliminary proof-of-concept work adds new knowledge to the field and this work should be extended by evaluating both morphologic and metabolic markers of viability. The alamarBlue® technology uses metabolism as a proxy for parasite death along with absence of motility, thereby reducing the risk of overcalling parasite death and underestimating the MIC. Additional markers of viability should be explored further. Finally, given the bipartite life cycle of the parasite, we must acknowledge that drug susceptibility testing profiles of cultured promastigotes may not approximate those exhibited by the amastigote form in mammalian hosts.

## Conclusion

We have demonstrated as proof-of-concept that the Sensititre™ YeastOne™ YO9 plate could be potentially adapted for routine *Leishmania* spp. promastigote susceptibility testing in a clinical microbiology laboratory. Our observation of metabolic and microscopic markers of parasite death along a standardized scale of drug concentrations may be a useful and objective complement to conventional epidemiological and clinical predictors of therapeutic response. Moreover, if validated, would likely inform clinical decision-making by risk stratifying patients according to the susceptibility profile of their infecting strain of *Leishmania*. Future testing of both promastigotes and amastigotes is warranted as well as susceptibility to FZ at higher concentrations of drug beyond the scope of the Sensititre™ YeastOne™ YO9 plate. Customization of the plate may provide the opportunity to evaluate higher concentrations of FZ that might have efficacy against *Leishmania* spp., given that all isolates in this study were resistant to FZ at ≥256 μg/mL.

## Data Availability

All data generated or analyzed during this study are included in this published article.

## Abbreviations

AB: Amphotericin B
AST: Antimicrobial susceptibility testing
ATCC®: American Type Culture Collection
BLAST: Basic Local Alignment Search Tool
CL: Cutaneous leishmaniasis
CLSI: Clinical & Laboratory Standards Institute
CPB: Cysteine proteainse B
FZ: Fluconazole
GCP: Good clinical practice
GLP: Good laboratory practice
HI-FBS: Heat-inactivated fetal bovine serum
HSP70: Heat shock protein 70
ITS1: Internal transcribed spacer 1
ITS2: Internal transcribed spacer 2
MIC: Minimum inhibitory concentration
ML: Mucosal leishmaniasis
N: Number of replicates
PCR: Polymerase chain reaction
RFLP: Restriction fragment length polymorphism
RPMI: Roswell Park Memorial Institute
